# Neuroprotective Drugs in Infants With Severe Congenital Heart Disease: A Systematic Review

**DOI:** 10.3389/fneur.2018.00521

**Published:** 2018-07-03

**Authors:** Raymond Stegeman, Kaya D. Lamur, Agnes van den Hoogen, Johannes M. P. J. Breur, Floris Groenendaal, Nicolaas J. G. Jansen, Manon J. N. L. Benders

**Affiliations:** ^1^Department of Neonatology, University Medical Center Utrecht, Utrecht University, Wilhelmina Children's Hospital, Utrecht, Netherlands; ^2^Department of Pediatric Cardiology, University Medical Center Utrecht, Utrecht University, Wilhelmina Children's Hospital, Utrecht, Netherlands; ^3^Department of Pediatric Intensive Care, University Medical Center Utrecht, Utrecht University, Wilhelmina Children's Hospital, Utrecht, Netherlands

**Keywords:** infant, congenital heart disease, cardiac surgery, cardiopulmonary bypass, neuroprotective drugs, brain injury, neurodevelopmental outcome

## Abstract

**Background:** Perinatal and perioperative brain injury is a fundamental problem in infants with severe congenital heart disease undergoing neonatal cardiac surgery with cardiopulmonary bypass. An impaired neuromotor and neurocognitive development is encountered and associated with a reduction in quality of life. New neuroprotective drugs during surgery are described to reduce brain injury and improve neurodevelopmental outcome. Therefore, our aim was to provide a systematic review and best-evidence synthesis on the effects of neuroprotective drugs on brain injury and neurodevelopmental outcome in congenital heart disease infants requiring cardiac surgery with cardiopulmonary bypass.

**Methods:** A systematic search was performed in PubMed, Embase and the Cochrane Library (PRISMA statement). Search terms were “infants,” “congenital heart disease,” “cardiac surgery,” “cardiopulmonary bypass,” and “neuroprotective drug.” Data describing the effects on brain injury and neurodevelopmental outcome were extracted. Study quality was assessed with the Cochrane Risk of Bias Tool. Two reviewers independently screened sources, extracted data and scored bias. Disagreements were resolved by involving a third researcher.

**Results:** The search identified 293 studies of which 6 were included. In total 527 patients with various congenital heart diseases participated with an average of 88 infants (13–318) per study. Allopurinol, sodium nitroprusside, erythropoietin, ketamine, dextromethorphan and phentolamine were administered around cardiac surgery with cardiopulmonary bypass. Allopurinol showed less seizures, coma, death and cardiac events in hypoplastic left heart syndrome (HLHS) infants (OR: 0.44; 95%-CI:0.21–0.91). Sodium nitroprusside resulted in lower post cardiopulmonary bypass levels of S100ß in infants with transposition of the great arteries after 24 (*p* < 0.01) and 48 (*p* = 0.04) h of treatment. Erytropoietin, ketamine and dextromethorphan showed no neuroprotective effects. Phentolamine led to higher S100ß-levels and cerebrovascular resistance after rewarming and at the end of surgery (both *p* < 0.01). Risk of bias varied between studies, including low (sodium nitroprusside, phentolamine), moderate (ketamine, dextromethorphan), and high (erytropoietin, allopurinol) quality.

**Conclusions:** Allopurinol seems promising for future trials in congenital heart disease infants to reduce brain injury given the early neuroprotective effects in hypoplastic left heart syndrome infants. Larger well-designed trials are needed to assess the neuroprotective effects of sodium nitroprusside, erytropoietin, ketamine and dextromethorphan. Future neuroprotective studies in congenital heart disease infants should not only focus on the perioperative period, however also on the perinatal period, since significant brain injury already exists before surgery.

## Introduction

### Rationale

Congenital heart disease (CHD) is the most common congenital malformation with an incidence varying from 4 to 50 per 1,000 live births (1). The incidence of severe forms of CHD—severely ill patients presenting in the newborn period or early infancy—is about 6 per 1,000 live births, including infants with transposition of the great arteries (TGA), univentricular heart physiology (UVH), aortic arch anomalies, tetralogy of Fallot (ToF), and large ventricular septal defects (VSD) undergoing cardiac surgery with cardiopulmonary bypass (CPB) (1). The survival of infants with severe CHD until adulthood has increased substantially to almost 90% during the last decades as a result of improved surgical procedures and intensive care (2). However, delayed brain development, brain injury and related long-term neurodevelopmental impairments are relevant problems in infants with severe CHD, indicating the urgent need for neuroprotective drugs. Altered cerebral circulation and reduced cerebral oxygenation already starts before birth and is associated with impaired brain growth in fetuses and neonates with severe CHD (3–5). The delayed brain development “*in utero*” increases the vulnerability for hypoxic-ischemic brain injury in postnatal life. The periods around birth and cardiac surgery with CPB are the most critical periods for the occurrence of brain injury (6–8). Early postnatally and preoperatively magnetic resonance imaging (MRI) of the brain shows injury in up to 63% of the infants with severe CHD (6, 7). After cardiac surgery with CPB up to 78% shows additional brain injury on MRI (8). Most common forms of brain injury seen in severe CHD infants are white matter injury (WMI) and focal infarctions of the gray matter, which are known to be caused by hypoxic-ischemic events (5, 8). Hypoxia causes excessive production of excitotoxins with overactivation of the N-methyl D-aspartate (NMDA-) receptor and calcium influx into neurons leading to cell damage and the release of pro-radicals and increased levels of xanthine. Upon reperfusion and reoxygenation reactive oxygen (“oxidative stress”) and nitrogen species are formed. This chain of events activates the inflammatory pathway with increased formation of pro- and anti-inflammatory cytokines resulting in inappropriate apoptosis and further brain injury. In addition neurotrophic factors are downregulated leading to diminished recovery of brain injury (9). Important consequences of brain injury in severe CHD infants are longterm neuromotor (standard deviation (SD) −1.5) and cognitive (SD −0.65) impairments (10), with even lower scores in infants with syndromic disorders (11). At school age, language disorders (20–30%), behavioral problems (20–40%), learning difficulties (30–50%) (12, 13) and impairments in executive functions are common (14). Therefore, it is of great importance to find ways to reduce hypoxic-ischemic brain injury and improve neurodevelopmental outcome (NDO) in this vulnerable population. A number of diagnostic and therapeutic neuroprotective strategies have been investigated. Most of these strategies focus especially on the period around cardiac surgery with CPB, including neuromonitoring with (amplitude integrated) electroencephalography, transcranial Doppler ultrasound and near-infrared spectroscopy and perfusion techniques as deep hypothermic circulatory arrest (DHCA), low flow CPB, and regional cerebral perfusion (15). Only avoidance of extreme hemodilution during hypothermic CPB (hematocrit level above 24%) is recommended. A low hematocrit strategy (mean 21.5%, SD 2.9) showed worse perioperative and neurodevelopmental outcomes in comparison to a higher hematocrit strategy (mean 27.8%, SD 3.2), as was indicated by higher lactate levels post-CPB (*p* = 0.03) and lower scores on psychomotor developmental index (82 vs. 90, *p* < 0.01) (16, 17). Some procedures or treatments are reasonable to consider, including deep hypothermia during CPB (18), avoiding hypoglycemia perioperatively (19), and postoperative normothermia (20). However, currently there is limited evidence for the effectiveness of the majority of the investigated neuroprotective strategies (15). The cascade leading to brain injury provides several pharmaceutical targets to intervene. Drugs that antagonize the NMDA-receptor, prevent oxidative stress, suppress the inflammatory response or upregulate neurotrophic factors could play a significant neuroprotective role in infants with severe CHD, both early postnatally, as well as perioperatively (9, 21). Currently, no standard neuroprotective drugs are used in infants with severe CHD. In light of the appearance of potential new neuroprotective drugs this systematic review appears to be useful.

### Objectives

The aim was to provide a systematic review and best-evidence synthesis on the effects of neuroprotective drugs on brain injury and neurodevelopmental outcome in congenital heart disease infants requiring cardiac surgery with cardiopulmonary bypass.

### Research question

Which neuroprotective drugs have been studied in infants with severe congenital heart disease and what is the evidence of the effects of these agents on brain injury and neurodevelopmental outcome?

## Methods

### Study design

A systematic review was performed following the steps of the Preferred Reporting Items for Systematic Reviews and Meta-Analyses (PRISMA) statement (22).

### Inclusion and exclusion criteria

Studies reporting on the effects of neuroprotective drugs on brain injury and/or NDO in infants with severe CHD requiring cardiac surgery with CPB were included. Gestational age at birth was restricted to at least 35 weeks (near-term and term) and patients with syndromic or genetic disorders were excluded. Reviews, studies that investigated other neuroprotective strategies than neuroprotective drugs and studies not written in English language were excluded. No restriction was set on the years of publication of the articles identified.

### Search strategy

The main search terms were infants, congenital heart disease, cardiac surgery, cardiopulmonary bypass and neuroprotective drug. Besides title and abstract, MESH terms were used for all search terms (Table 1).

**Table 1:** Search strategy.

**MESH**	**TiAb**
Infant	Neonat^*^ Infant^*^ Newborn^*^ Child^*^
Heart defects, congenital	Congenital heart disease, congenital heart defect Aortic coarctation, coarctation of the aorta Hypoplastic left heart syndrome Transposition of the great vessels, transposition of the great arteries
Cardiac surgical procedures	Cardiac surgery Heart surgery Coarctectomy Norwood Arterial switch
Cardiopulmonary bypass	Cardiopulmonary bypass
**AND**	
Neuroprotective agents	Neuroprotect^*^

* *Truncation symbol was used to find terms with other endings or an alternative spelling*.

### Data sources, studies selections and data extraction

PubMed, Embase and The Cochrane Library were searched in the period from inception to May 30th 2017 to identify suitable articles. Scopus and Web of Science were searched for additional articles through reference screening. Citations of included articles were manually screened for relevant articles. After removal of duplicates, articles were screened on title and abstract and records not matching the inclusion criteria were excluded. The remaining articles were assessed full-text for eligibility. After exclusion of full-text articles not answering the research question, a decision was made of studies to be included in the final systematic review. The study characteristics and relevant findings of the included studies were recorded on a data extraction file. The following study characteristics were extracted: study design, number of infants, type of CHD, age at cardiac surgery with CPB, drug, moment of administration, dose and mode of administration, outcome and outcome assessment. The selection of studies and data extraction was performed independently by two researchers (RS, KDL) and any disagreements were resolved by involving a third researcher (NJGJ, AvdH).

### Data analysis

The Cochrane Risk of Bias Tool was used to assess the methodological quality of the included studies (23, 24). The included studies were assessed on random sequence generation (selection bias), allocation concealment (selection bias), blinding of participants/personal (performance bias), blinding of outcome assessors (detection bias), incomplete outcome data (attrition bias), selective reporting (reporting bias), and other forms of bias. The risk of bias and overall quality of the studies was assessed independently by two researchers (RS, KDL) and disagreements were resolved by involvement of a third researcher (NJGJ, AvdH). A total of six forms of bias were scored as low or high risk. Studies were considered of high quality when at least 5 forms of bias scored low risk, of moderate quality when 4 forms of bias scored low risk and of low quality in case 3 or less forms of bias scored low risk.

### Best evidence synthesis

A best-evidence synthesis was performed since the outcome measures of the included studies were too heterogeneous for a meta-analysis. Both the outcome (Table 2) and the quality (**Figure 2**) of the included studies were taken into account.

**Table 2:** Study characteristics, relevant findings, and risks of bias.

**Study**	**Design no**.	**CHD age**	**Drug moment Dose and mode**	**Outcome (Assessment)**	**Relevant findings**	**Risks of bias summarized**
Abdul–Khaliq et al. (30)	PC *N = 53*	TGA *0.36 M*	**Sodium nitroprusside** *During and after surgery 1–5 μg/kg/min IV*	Brain injury (*S100ß*)	Lower post-CPB levels S100ß after 24 h (2.0 vs. 2.9 μg/L; *p* = 0.0009) and 48 h (1.0 vs. 1.8 μg/L; *p* = 0.04) of treatment.	High
Andropoulos et al. (26)	RCT *N = 59*	TGA, HLHS, AAA * < 30 D*	**Erythropoietin** *Before and after surgery 1,000 or 500 U/kg IV*	Brain injury (*MRI*) NDO (*BSITD-III, mean 100, SD 15*)	No difference in brain injury, clinical events and death. No difference in NDO composite scores at 12 M, including cognitive (101 vs. 106; *p* = 0.19), language (89 vs. 92; *p* = 0.33), and motor (90 vs. 93, *p* = 0.51).	Low
Bhutta et al. (28)	RCT *N = 24*	VSD *5.4 M*	**Ketamine** *Before and after surgery 2 mg/kg IV*	Brain injury (S*100ß, NSE, MRI/S*) NDO (*BSITD-II)*	No differences in S100ß, NSE at the end to 48 h after surgery. No structural abnormalities on pre- and post-operative MRI. Decrease in glutaminate/glutamate (*p* = 0.006) on postoperative MR-spectroscopy. No differences in MDI, PDI pre- and 2/3 weeks post-operative.	Moderate
Clancy et al. (27)	RCT *N = 318*	HLHS, non-HLHS *5.6 D*	**Allopurinol** *Before, during, after surgery 5, 10, and 20 mg/kg IV*	Brain injury (*clinical seizures, coma*), Death, cardiac events	No treatment effect on primary outcome: death, clinical seizures, coma. Lower event rate death, seizures, coma, cardiac events in HLHS (38 vs. 60%; OR 0.44; 95% CI 0.21–0.91). Less endpoint events in HLHS surgical survivors (event free 85 vs. 55%; *p* = 0.002). Fewer seizures-only (4 vs. 18%; *p* = 0.05) and cardiac-only (4 vs. 20%; *p* = 0.03) events in HLHS.	Low
Gazzolo et al. (31)	RCT *N = 60*	TOF, VSD, TGA, AS *124–128 D*	**Phentolamine** *Before cooling and rewarming 0.2 mg/kg IV*	Brain injury (*S100ß)* Cerebral hemodynamics (*MCA-PI*)	S100ß higher after rewarming (3.53 vs. 1.58 μg/L; *p* < 0.001) and at the end of surgery (2.95 vs. 0.79 μg/L; *p* < 0.001). Higher MCA-PI values at the end of surgery (1.83 vs. 1.22; *p* < 0.01).	High
Schmitt et al. (29)	RCT *N = 13*	VSD, TOF *3–36 M*	**Dextromethorphan** *Before and after surgery 36–38 mg/kg/day NG-tube*	Brain injury (*MRI, NSE*) Cerebral activity (*EEG*) NDO (*Griffiths, mean 100)*	Less pronounced ventricular enlargement (not significant). Increase in white matter lesions in 2 placebo-treated children only. NSE-level in CSF similar. Less often sharp waves on postoperative EEG (7/7 vs. 2/6; *p* = 0.02). Developmental quotients pre- (98 vs. 95), post-operative (92–85), at 3 months (92 vs. 93) similar.	Moderate

*AAA, Aortic Arch Anomalies; AS, Aortic Stenosis; BSITD, Bayley Scales of Infant and Toddler Development; CHD, Congenital Heart Disease; CSF, CerebroSpinal Fluid; EEG, electroenchephalography; HLHS, Hypoplastic Left Heart Syndrome; MCA-PI, Middle Cerebral Artery Pulsatility Index; MDI, Mental Developmental Index; MRI, Magnetic Resonance Imaging; MRS, Magnetic Resonance Spectroscopy; NDO, Neuro Developmental Outcome; NG, Naso Gastric; NIRS, Near-Infrared Spectroscopy; NSE, Neuron Specific Enolase; PC, Prospective Cohort; PDI, Psychomotor Developmental Index; RCT, Randomized Controlled Trial; SD, standard deviation; TOF, Tetralogy of Fallot; TGA, Transposition of the Great Arteries; VSD, Ventricular Septal Defect*.

## Results

### Flowchart

**Figure 1:**
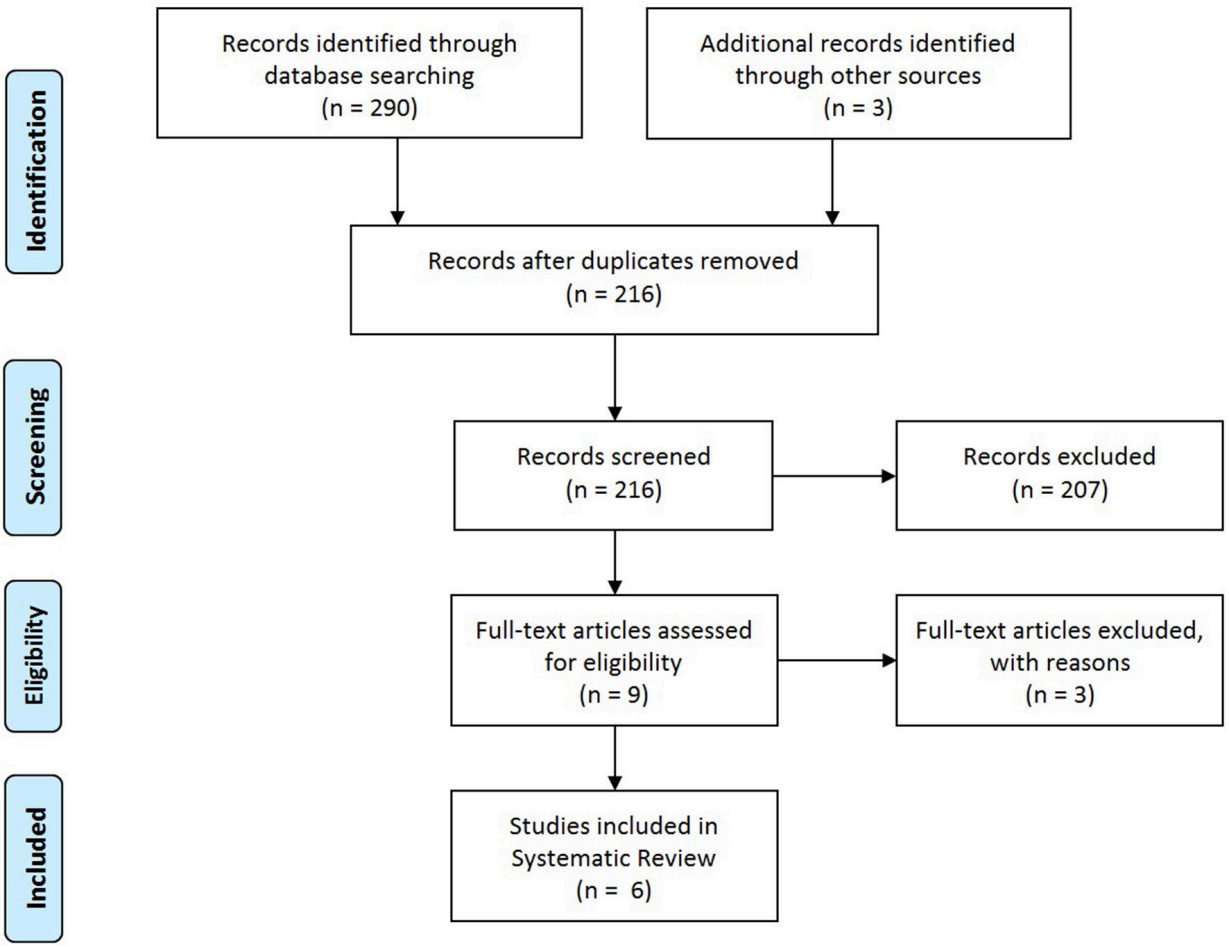
PRISMA flow diagram; adapted from Moher et al. (22).

The search identified 293 records, including 290 through database searching (Pubmed *n* = 83, Embase, *n* = 194, Cochrane *n* = 13) and 3 by reference screening. After removing duplicates, 216 studies were screened on title and abstract. Of these, 207 were excluded since they met the exclusion criteria and/or did not met the inclusion criteria. Nine full-text articles were assessed for eligibility of which 3 were excluded because only the abstract was available (*n* = 1) or no answer was given to the research question (*n* = 2). Finally, 6 studies were included in this systematic review (Figure 1: adapted from Moher et al. (22)).

### Study characteristics

A total of 5 randomized controlled trials (RCTs) and 1 prospective cohort study were eligible for the review comprising a total of 527 patients (ranging from 13 to 318 per study). The age of the included infants with CHD in these studies, ranged from < 30 days to 36 months. Various cardiac defects as TGA, UVH, aortic arch anomalies, VSD, and TOF were included. Six neuroprotective drugs were investigated: sodium nitroprusside (SNP), erythropoietin (EPO), ketamine, allopurinol, phentolamine, and dextromethorphan. All were administered intravenously around cardiac surgery with CPB, with the exception of dextromethorphan which was given orally by nasogastric tube. The drugs were compared with placebo, except SNP which was compared with standard care. Various outcome measures of brain injury (S100ß, neuron specific enolase, MRI, clinical seizures, coma) and NDO (Bayley II/III, Griffiths) were taken into account (Table 2). S100ß and neuron specific enolase are released in blood in the setting of brain injury from glial cells and neurons respectively, and are related to brain injury by MRI and early neurodevelopmental outcomes (25).

### Methodological analysis

**Figure 2:**
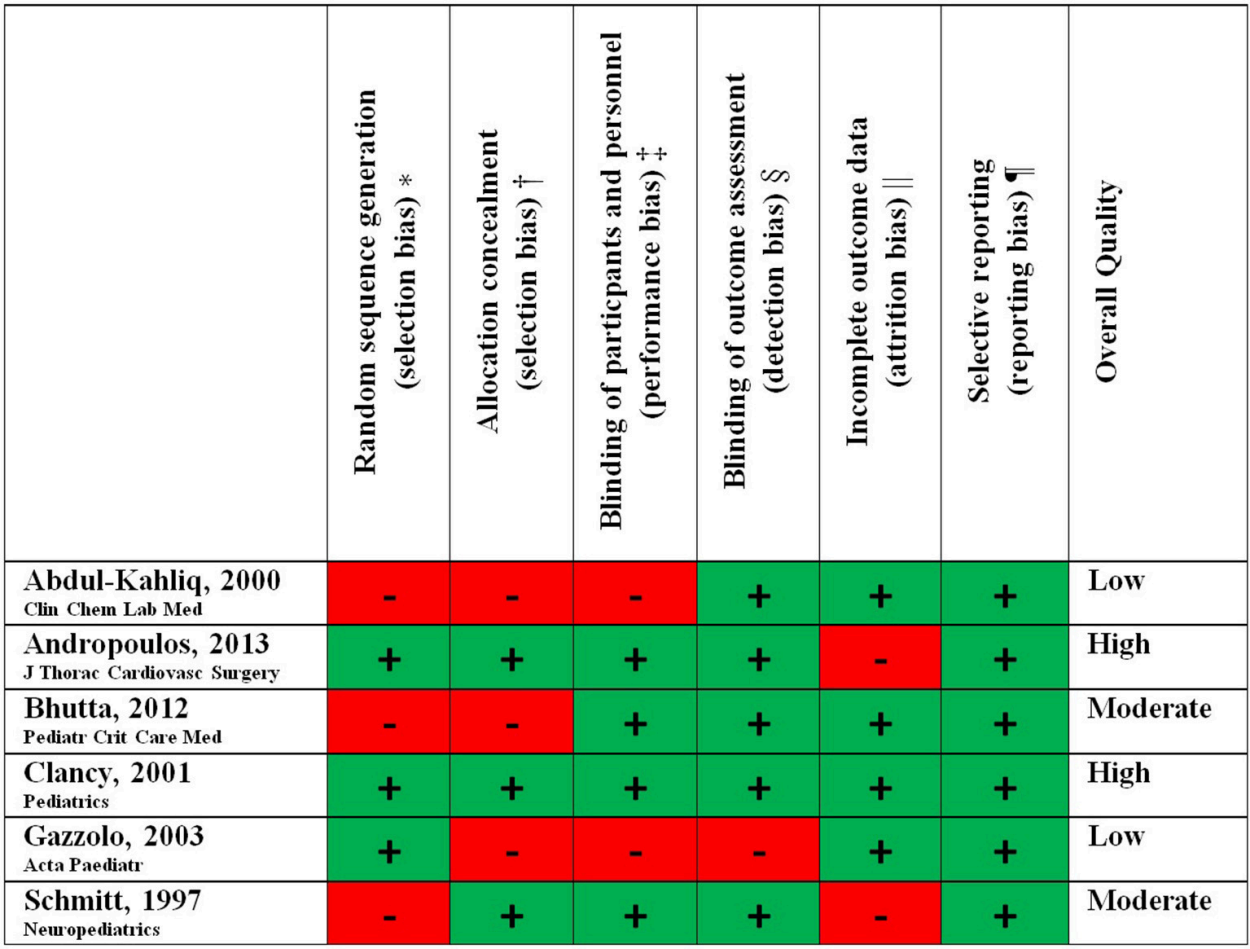
Methodological study quality. ^*^Biased allocation to interventions due to inadequate generation of a randomized sequence. †Biased allocation to interventions due to inadequate concealment of allocations prior to assignment. ‡Knowledge of the allocated interventions by participants and personnel during the study. ^§^Knowledge of the allocated interventions by outcome assessors. ^||^Amount, nature or handling of incomplete outcome data. ¶Selective outcome reporting.

The overall methodological quality of the included studies was analyzed with the Cochrane Risk of Bias Tool (23, 24) There were 2 studies of high (26, 27) *(EPO, allopurinol)*, 2 studies of moderate (28, 29) *(ketamine, dextromethorpan)* and 2 studies of low (30, 31) *(SNP, phentolamine)* methodological quality (Table 2, Figure 2).

### Sodium nitroprusside (SNP)

In 2000, Abdul-Khaliq et al. published a prospective cohort study in which they evaluated the effect of continuous treatment with the nitric-oxide (NO-) liberator SNP on the brain injury marker S100ß in 53 neonates after cardiac surgery with hypothermic CPB for TGA. SNP was infused (1–5 microgram per kilogram bodyweight per minute depending on the hemodynamic status) after the induction of anesthesia, and during and after the termination of CPB for 2 days. SNP treated neonates (*n* = 25, 0.37 months) had significantly lower levels of S100ß 24 h (2.0 vs. 2.9 μg/L, *p* = 0.009) and 48 h (1.0 vs. 1.8 μg/L, *p* = 0.04) after surgery in comparison to non-treated infants (*n* = 28, 0.32 months). S100ß levels 24 h after surgery normalized to preoperative values in the SNP treated neonates, however remained significantly high in the non-treated infants (*p* = 0.01) (30). This study showed that continous low-dose treatment with the NO liberator SNP was safe and decreased the release of S100ß into the blood stream after corrective cardiac surgery with CPB for TGA infants. However, the overall quality of the study was low with both a high risk on selection bias as well on performance bias. Patients were not randomized and the cross-clamping time (minutes) at baseline was significantly higher in the SNP-treated group (median 98, range 50–174) compared to the standard-treated group (78, 67–114) (*p* = 0.004). Furthermore, parents of participating children and (treating) physicians were not blinded and were aware of the treatment-group (30).

### Erythropoietin (EPO)

In 2013 Andropoulos et al. determined the anti-apoptotic, anti-excitatory and anti-inflammatory effects of EPO on brain injury and NDO at 12 months in a phase I/II safety and efficacy randomized, blinded, placebo-controlled trial. Fifty-nine neonates (age < 30 days) with TGA, hypoplastic left heart syndrome (HLHS) or aortic arch anomalies received 3 intravenous doses of EPO (500 or 1,000 U/kg) or placebo before and after hypothermic CPB. There were no differences between treated infants and controls in clinical events (as cardiac arrest, the need of extracorporeal membrane oxygenation and seizures), physical neurological examination preoperatively and before discharge, and mortality. Brain MRI was performed immediate preoperatively and postoperatively (at 7–10 days after surgery). MRI-scans were evaluated by blinded pediatric neuroradiologists and assessed on mild/moderate/severe white matter injury, intraparenchymal infarction, intraparenchymal or intraventricular hemorrhage, and sinovenous thrombosis. No differences in rate and severity of preoperative and postoperative brain injuries were observed. Neurodevelopmental testing with the Bayley Scales of Infant Development (BSITD-III, mean value 100, SD 15) at 12 months were not significantly different between the treated infants and controls, including cognitive (101 vs. 106; *p* = 0.19), language (89 vs. 92; *p* = 0.33), and motor composite scores (90 vs. 92, *p* = 0.51). This study showed no significant differences in safety profile (including brain injury) and NDO after perioperative EPO or placebo administration. The overall methodological quality of the study was high with only a high risk on attrition bias by incomplete outcome data (loss to follow-up 21%) (26).

### Ketamine

In 2012, Bhutta et al. studied the effect of the anesthetizing, anti-inflammatory and anti-excitotoxic non-competitive NMDA receptor antagonist ketamine on S100ß, neuron specific enolase (NSE), brain injury (MRI and proton MR-spectroscopy) and NDO (BSITD-II) in a pilot RCT. Twenty-four infants with a mean age of 5.4 months received 2 mg/kg intravenous ketamine (*n* = 13) or placebo (*n* = 11) before CPB for VSD repair. Postoperative MR-spectroscopy showed a significant decrease in choline a marker of demylination and glutamate plus glutamine/creatine a marker of excitotoxic neuronal and glial cell death in frontal white matter of the brain. There were no structural abnormalities on pre- and post-operative MRI and no differences in S100ß and NSE at the end to 48 h after surgery. Preoperative and postoperative (2–3 weeks after surgery) BSITD-II scores showed no significant differences in mental (MDI) and psychomotor developmental index (PDI). This study did not find neuroprotective effects of ketamine on brain injury or NDO. The overall methodological quality of this study was moderate, since there was a high risk on selection bias. Significant differences in several clinical parameters (such as intraoperative cooling) were present at baseline and no procedure for allocation concealment was described (28).

### Allopurinol

In 2001, Clancy et al. investigated in a single center, randomized, placebo-controlled, blinded trial the effects of the free radical scavenger allopurinol on clinical seizures, coma, death and cardiac events in infants undergoing cardiac surgery with DHCA. Cardiac events were defined as periods of acute, severe cardiorespiratory deterioration necessitating immediate resuscitation such as chest massage, defibrillation, and acute boluses of inotropics. A total of 318 HLHS (*n* = 131) and non-HLHS (*n* = 187, other forms of CHD than HLHS) infants (mean age 5.6 days) received intravenous allopurinol 5–20 mg/kg or placebo before, during and after surgery. There was no significant difference in the primary endpoint (death, clinical seizures, coma) between allopurinol treated infants and controls. However subgroup analysis showed that allopurinol in comparison to placebo resulted in a lower event rate of clinical seizures, coma, death and cardiac events in HLHS-infants (38 vs. 60%; OR 0.44; 95%-CI 0.21–0.91), but not in non-HLHS infants (30 vs. 27%; OR 1.17; 95%-CI 0.61–2.25). There were significantly less clinical seizures (4 vs. 18%, *p* = 0.05) and cardiac events (4 vs. 20%, *p* = 0.03) after allopurinol vs. placebo treatment in the HLHS-group, but there was no difference in mortality. In HLHS survivors allopurinol showed less endpoint events (clinical seizures, coma or cardiac event) compared to placebo (event free 85 vs. 55%, *p* = 0.002). Safety profile was similar between both groups. This study showed significant neurocardiac protection in HLHS infants. The overall methodological quality was high since there were no risks of bias identified (27).

### Phentolamine

In 2003, Gazzolo et al. studied in a RCT the effect of the vasodilating non-selective catecholamine receptor blocker phentolamine on S100ß-levels and middle cerebral artery pulsatility index (MCA-PI) before, during and after surgery. Sixty patients (age 124–128 days) undergoing CHD surgery for TOF, (multiple) VSD(s), TGA or aortic stenosis received 0.2 mg/kg phentolamine (*n* = 30) or placebo (*n* = 30) before the cooling and rewarming phases of CPB. Cooling and rewarming times were shorter in the phentolamine-treated group (*p* < 0.01). Phentolamine treated infants had significantly higher levels of S100ß after rewarming (3.53 vs. 1.58 μg/L, *p* < 0.001) and at the end of surgery (2.95 vs. 0.79 μg/L, *p* < 0.001) than placebo-treated infants. The cerebrovascular resistance (MCA-PI values) was also significantly higher at the end of surgery in phentolamine treated infants (1.83 vs. 1.22, *p* < 0.01). This study showed that phentolamine administration to shorten the cooling and rewarming phases of CPB was correlated with increased brain damage and cerebrovascular resistance. The overall methodological quality was low since there was a high risk on selection, performance and detection bias. No procedure for allocation concealment was described and parents, study personnel and outcome assessors were aware and not blinded for treatment group respectively (31).

### Dextromethorphan

In 1997, Schmitt et al. determined the effect of the non-competitive NMDA antagonist dextromethorphan in a pilot RCT on brain injury (MRI, NSE), cerebral activity (electroencephalography or EEG) and neurodevelopmental outcome (Griffiths). Thirteen infants and children (3–36 months) with VSD or TOF received dextromethorphan 36–38 mg/kg/day (*n* = 6) or placebo (*n* = 7) by nasogastric tube before and after surgery with CPB. Pre- and post-operative MRI showed less ventricular enlargement (non-significant) in the dextromethorphan group. Periventricular white matter lesions were only seen in 2 placebo-treated children. Levels of NSE were not increased in both groups. Postoperative EEG showed significant less sharp waves in the dextromethorphan vs. placebo-group (2 vs. 7, *p* = 0.02). Griffiths developmental quotients (normal value 100) before the operation (mean 98 vs. 95), at hospital discharge (92 vs. 85) and after 3 months (92 vs. 93) were similar in both groups. Adverse effects were not observed. This study showed no significant neuroprotective effects of dextromethorphan on brain injury and early NDO (29). The study quality was moderate with a high risk on both selection and attrition bias. ToF was more often diagnosed in the placebo group and some outcome data were incomplete.

### Best evidence synthese

The current evidence of the neuroprotective effects of SNP (30), EPO (26), ketamine (28), and dextromethorphan (29) is too insufficient, due to the quality of these studies, to make any recommendation for clinical usage at this moment. Allopurinol is the only drug that may be considered as is shown in a high quality study. However the effect on structural brain abnormalities and longterm NDO is not well-established and requires further investigation (27). Phentolamine should not be recommended given the potential neurotoxic effects as shown in a study of low quality (31).

## Discussion

This systematic review assessed the available evidence of potential neuroprotective drugs on brain injury and/or NDO in infants with severe CHD requiring cardiac surgery with CPB. The current evidence was limited as only 6 different drugs were studied in this population. SNP and allopurinol showed potential neuroprotective effects. EPO, ketamine and dextromethorphan showed no neuroprotective effects whereas phentolamine showed neurotoxic effects. However, the evidence of these studies was not sufficient enough to make any recommendation for usage in clinical practice. First, larger well-designed trials are needed, for which allopurinol is a promising candidate.

Five studies were placebo-controlled (pilot) RCTs, while the study of Abdul Kahliq et al. (30) was the only one which prospectively compared SNP with standard treatment. The sample sizes were limited (*n* = 13–60) and only the study of Clancy et al. (27) studied allopurinol in a large number of 318 infants. A heterogeneous group of cardiac defects was studied. The age of the study participants varied and was not only limited to neonates. Ketamine (28) and phentolamine (31) were studied in infants whereas dextromethorphan (29) was studied in young children up to 36 months. All studies focused on the period around cardiac surgery with CPB and not (also) on the vulnerable perinatal and early postnatal period. Brain injury *(clinical seizures, coma, S100ß, NSE, Doppler, EEG, MRI)* and/or NDO *(BSITD, Griffiths)* were measured in different ways, making it not possible to compare the outcomes of the studies included.

The study of Abdul-Kahliq et al. (30) indicates that continues low-dose treatment with the NO-liberator SNP during and after surgery for TGA may give delayed neuroprotection by reducing astroglial cell activation and disintegration of the blood-brain barrier (“oxidative stress”). However, the overall methodological quality of the study was low. Patients were not randomized and the cross-clamping time was significantly higher in the SNP-treated group compared to the standard-treated group. This may have led to an underestimation of the neuroprotective effects of SNP in this study. Therefore, a well-designed study seems needed to evaluate the true effects of SNP on structural brain abnormalities and longterm NDO. Previous *in vitro* studies showed protective effects of nitric oxide on blood-brain barrier after hypoxia reoxygenation mediated injury, by effectively scavenging reactive oxygen species (32).

Andropoulos et al. (26) found no significant neuroprotective effects of perioperative EPO administration on NDO at 12 months. Despite the high quality of the study the power was not sufficient to demonstrate a NDO difference. In addition, the change in EPO dosage (from 1,000 to 500 units/kg) by the FDA during the study may have led to levels that may not be neuroprotective. Because of these limitations and the promising results of EPO on neurodevelopment-related outcomes in neonates with hypoxic-ischemic encephalopathy (HIE) (33, 34) and very low birthweight infants (35), a larger RCT would be required to definitively address the neuroprotective effects of EPO in this CHD population. In animal and *in vitro* models EPO protects the brain against cerebral insults and cell death by anti-excitatory, anti-inflammatory and anti-apoptotic mechanisms (36).

Bhutta et al. (28) found no significant neuroprotective effects of pre-CPB administration of Ketamine on structural brain injury and early postoperative NDO. However, the anti-excitotoxic and anti-inflammatory effects (NMDA-antagonism) of ketamine led to a significant decrease in myelin breakdown and cell death mediated excitotoxicity in the frontal white matter of the brain, as was indicated by functional MR spectroscopy. This study was of moderate quality, had a small sample size (*n* = 24) and showed significant baseline differences despite randomization. Apart from the results of this study doubts have recently emerged over the safety of anesthetics, including ketamine, in recent neonatal animal studies and children under the age of 3 (37, 38). Large doses of Ketamine given repeatedly or as continuous infusion for prolonged periods can induce apoptotic cell death (37). Currently, trials are underway to investigate these dose-related and exposure-time effects of anesthesia on longterm NDO in young children. Further research with ketamine in CHD neonates should be postponed, until the results of these trials are known (38).

Clancy et al. (27) showed that perioperative allopurinol administration was safe and provided a neurocardiac protective effect (death, coma, clinical seizures, cardiac events) in higher-risk HLHS infants. The methodological quality of this study was high given the low risks of bias. The neuroprotective effects in HLHS-infants were suggested by significantly fewer clinical seizures in the allopurinol vs. placebo-group. The occurrence of perioperative seizures is an early sign of new brain injury and associated with worse neurodevelopmental outcome (39). However, the effects of the xanthine-oxidase inhibitor allopurinol on amplitude integrated EEG and structural brain injury with pre- and post-operative MRI and longterm NDO were not assessed. The definitive neuroprotective effect of allopurinol in the CHD population should be demonstrated by including these study procedures in a future high quality study. The possible neuroprotective effects of allopurinol are based on several preclinical studies in rats, piglets and sheep and clinical studies in neonates with HIE (40). In neonates with HIE beneficial effects were found in three small studies (41–43) in which allopurinol was administered postnatally and a pilot (44) and multicenter (45) study in which allopurinol was administered antenatally. Longterm NDO was only beneficial after postnatal allopurinol treatment in infants with moderate HIE (46). The ALBINO-trial (NCT03162653) will investigate the neuroprotective effect of early postnatal allopurinol as add-on therapy to hypothermia on NDO in HIE-neonates.

The study of Gazollo et al. (31) indicates that phentolamine administration to shorten the cooling and rewarming phases of CPB is neurotoxic and increases brain damage. However, the study was of low quality since there was no blinding and no allocation concealment procedure was described. Notwithstanding the beneficial effects of the non-selective alpha-1, 2 receptor blocker phentolamine on the duration of CBP and surgery, it seems correlated with increased brain stress during CPB and should not be recommended. Other vasodilator agents used in this population should also be investigated on account of their possible undesired effects.

The study of Schmitt et al. (29) showed less abnormalities on EEG and MRI after perioperative administration of high-dose oral dextromethorphan. However, this moderate quality study was too small and there were significant dissimilarities between the treatment groups, making conclusions about possible neuroprotective effects of the NMDA-antagonistic properties of dextromethorphan at this time not possible. Further larger well-designed studies are encouraged because of the good resorption and tolerance of orally administered dextromethorphan.

Some explanations can be given for the limited number of RCTs that have been performed concerning the effects of neuroprotective drugs in the population of severe CHD infants. First, the need for neuroprotective drugs has only recently become clear, since we now know more about the immature brain development, perinatal/perioperative brain injury, and consequently longterm neurodevelopmental impairments within this specific population. In addition, infants with severe CHD are a relatively rare (low number of patients per center) and heterogeneous population (different cardiac defects, prenatal and postnatal diagnosis). Therefore, a large number of patients is needed to perform a RCT and many centers have to be involved using the same treatment (study drug vs. placebo) and strategies at their intensive care unit and during the perioperative setting. Lastly, often funding is first needed for these RCTs which is challenging to accomplish because of the hight costs.

In recent reviews of Robertson et al. and Hagberg et al. overviews were given of neuroprotective agents in animal models and a term newborns with perinatal brain injury by hypoxic-ischemic encephalopathy, intracranial hemorrhage and stroke. Besides allopurinol and EPO, also other agents as tetrahydrobiopterin (BH4), melatonin, topiramate, nitric oxide inhibitors, xenon, N-acetylcysteine (NAC), vitamins C and E, and stem cells were discussed (9, 47). Although the pathophysiology of brain injury is different in neonates with HIE, these drugs can also apply for infants with severe CHD in the future.

Finally, a number of recommendations are worth mentioning for future studies investigating the effects of neuroprotective drugs on brain injury and NDO in infants with severe CHD. (1) Well-designed RCTs with adequate sample sizes are needed taking into account the heterogeneity of the CHD population. (2) Drugs should be administered in the neonatal period, since the greatest neuroprotective effect can be expected in this phase. This is the most vulnerable period in which the brain develops the fastest. (3) Neuroprotective drugs should be administered in both the perinatal/early postnatal phase and perioperative period. Recent research showed that brain injury occurs during both of these vulnerable phases (6, 8). (4) Brain imaging with preoperative and postoperative MRI is an important study procedure to assess brain injury quantitatively. (5) Longterm neurodevelopmental follow-up (including measurements of executive functioning) is necessary as neurodevelopmental impairments become more pronounced as these children grow up (“grow in their deficits”).

## Author contributions

All authors have made a significant contribution to the manuscript. RS and KL performed the literature search, screened and selected the studies, extracted data, assessed risk of bias, drafted the initial manuscript, and approved the final manuscript as submitted. AvdH coordinated the literature search, screening and selection of studies, extraction of data and assessment on risk of bias, reviewed and revised the manuscript, and approved the final manuscript as submitted. JB conceptualized the study, reviewed and revised the manuscript, and approved the final manuscript as submitted. FG conceptualized the study, coordinated the selection of studies, reviewed and revised the manuscript and approved the final manuscript as submitted. NJ conceptualized the study, coordinated the literature search, screening and selection of studies, extraction of data and assessment on risk of bias, reviewed and revised the manuscript, and approved the final manuscript as submitted. MB conceptualized the study, edited the final manuscript, and approved the final manuscript as submitted.

### Conflict of interest statement

The authors declare that the research was conducted in the absence of any commercial or financial relationships that could be construed as a potential conflict of interest.
